# Evaluating the Incidence of Seizures and Its Relationship With Blood Glucose Levels in Tramadol Poisoning Patients Admitted to Imam Reza Hospital in Mashhad (Iran) From 2019 to 2020

**DOI:** 10.1155/jt/8335323

**Published:** 2025-02-01

**Authors:** Seyed Reza Mousavi, Faezeh Jafari, Anahita Alizade Ghamsari, Mina AkbariRad, Majid Khadem-Rezaiyan, Seyed Amirhossein Mousavi, Sadaf Sadat Rafati

**Affiliations:** ^1^Medical Toxicology Research Center, Faculty of Medicine, Mashhad University of Medical Sciences, Mashhad, Iran; ^2^Department of Internal Medicine, Faculty of Medicine, Mashhad University of Medical Sciences, Mashhad, Iran; ^3^Department of Community Medicine, Faculty of Medicine, Mashhad University of Medical Sciences, Mashhad, Iran

**Keywords:** blood glucose level, consciousness, poisoning, seizures, tramadol

## Abstract

**Background:** Drug poisoning is the most common type of poisoning in the world. The utilization of tramadol for the management of pain has been identified as a significant contributor to the incidence of poisoning cases. Tramadol poisoning can result in a range of neurological complications, including seizures and a decreased level of consciousness. Tramadol-induced seizures are frequently dose independent and manifest as generalized tonic-clonic seizures. The neurotoxic effects of tramadol are primarily manifested within the initial 24 h period following ingestion, with 84.6% of the seizures occurring within the first six hours. In addition, it has been documented that 15%–35% of the patients with tramadol poisoning have experienced seizures. The present study was undertaken to elucidate the clinical and paraclinical signs and symptoms observed in patients with tramadol poisoning and their correlation with the occurrence of seizures.

**Methods and Materials:** All patients hospitalized due to tramadol poisoning from October 2019 to September 2020 in the poisoning department of Imam Reza Hospital if they met the inclusion criteria were studied. The patients were divided into two groups with and without seizures. The occurrence of seizures was substantiated through the documentation of EMS personnel and the direct observation of the attending physician in the emergency room. Following admission, the patient's blood glucose level was quantified via a glucometer. A blood sample was also obtained for subsequent laboratory evaluation. In the event of any aberrations in blood glucose levels, a re-evaluation was conducted at one-hour intervals using a glucometer. All findings were analyzed using SPSS Version 25 statistical software. The level of significance was set at *p* < 0.05.

**Results:** A total of 163 patients were included in this study. In 94 patients (57.3%), some degree of consciousness loss and seizures occurred in 69 patients (42.1%). There was a significant relationship between the occurrence of seizures and the increase in blood glucose levels of patients (*p*=0.031). The findings indicated that 60% of the patients with blood glucose levels exceeding 140 mg/dL experienced seizures.

**Conclusion:** Seizures in tramadol poisoning may be related to the patient's blood glucose levels.

## 1. Introduction

Tramadol is one of the most common synthetic narcotic drugs in the world, with a high prevalence of poisoning cases [1]. Epidemiological studies have demonstrated that 69 individuals out of every 1000 individuals engage in the abuse of this substance. A greater proportion of tramadol abuse and poisoning cases are observed in young men in their third decade of life than in women. In terms of poisoning, the most prevalent route of administration is oral consumption, accounting for over 98% of the instances. The most common cause of poisoning is suicide attempts, with a reported prevalence of 52%–80% [2]. This combination is indicated for the short-term management of moderate to severe pain. It is also used as the first line of treatment in musculoskeletal pain and in patients in whom the use of NSAIDs is contraindicated or those who have pain resistant to oral analgesics [3]. Tramadol is an analog of 4-phenylpiperidine-codeine and acts as a weak agonistic effect on the *μ* receptor, and the reuptake inhibitory effects of serotonin and noradrenaline ultimately cause inhibitory effects on pain transmission in the spinal cord [4]. The excessive consumption of this substance has been linked to several adverse effects, including weakness and lethargy, restlessness, dizziness, headache, double vision, blurred vision, sweating, movement disorders, nausea, constipation, tachycardia, hypotension, convulsions, and coma [5]. Complications of the nervous system, including restlessness, decreased level of consciousness, and seizures are the most common manifestations of its overdose [2]. Other less common complications include cardiovascular symptoms (hypotension, sinus tachycardia, and cardiopulmonary arrest), respiratory symptoms (shortness of breath and severe allergic reactions) [6, 7], gastrointestinal disorders, sexual problems, serotonin syndrome [8], renal system disorders (acute kidney failure, interstitial nephritis, and hyponatremia), and musculoskeletal system disorders (rhabdomyolysis) [7, 9, 10]. Also, the inhibitory effect of tramadol on the absorption of serotonin and norepinephrine along with the increase in peripheral glucose consumption through the glutamate receptor (GLUT4) can lead to a severe decrease in blood sugar [11, 12]. Consequently, the administration of tramadol, even in diabetic patients, results in hypoglycemia [13]. In addition, some studies have documented the occurrence of hyperglycemia in patients [14]. It has been reported that the monoaminergic pathways involved in the analgesic effect of tramadol may also be involved in the development of hyperglycemia [15]. Nevertheless, serial monitoring of blood glucose levels is an effective method for the early detection and management of hypoglycemia and hyperglycemia in cases of overdose and poisoning with tramadol to reduce complications, particularly seizures [16]. Seizures are reported as a more serious neurological disorder associated with tramadol poisoning than with other opioids. In cases of tramadol overdose, the incidence of seizures is reported to range from 8% to 81%. The majority of seizures induced by tramadol occur within the initial 6 hours following ingestion. Seizures are typically tonic-clonic and unifocal. Several studies have demonstrated that convulsions can occur not only in the event of an overdose of tramadol but also in response to low therapeutic doses (50–100 mg) [2]. The amount and duration of tramadol consumption and simultaneous use of sedative or narcotic drugs along with factors such as previous diseases and history of family diseases, age, gender, and other demographic variables can affect the complications caused by tramadol consumption [17]. In recent years, tramadol poisoning has increased significantly in Iran and the world [18]. The sale of tramadol in pharmacies and the open market and the public's ignorance of the negative consequences of tramadol consumption are the main reasons for the increase in poisoning [4]. Considering that in many studies, decreased levels of consciousness and convulsions have been reported as the main side effects of tramadol, if there is a significant relationship between the clinical symptoms and the occurrence of these side effects, they can be prevented. The objective of this study was to examine the clinical and paraclinical signs and symptoms in patients who had been poisoned by tramadol, as well as to determine the relationship between these findings and the occurrence of seizures. Furthermore, given the numerous reports in recent studies indicating that electrocardiogram (ECG) alterations can predict the onset of seizures in patients [19–21], we sought to assess the impact of these changes on the incidence of seizures in individuals with tramadol poisoning.

## 2. Methods and Materials

The present study was designed for cross-sectional analysis. All patients admitted to Imam Reza Hospital's poisoning department in Mashhad due to tramadol poisoning from October 2019 to September 2020 were examined if they did not meet the exclusion criteria. The exclusion criteria comprised concurrent use of medications or other substances with tramadol, a previous medical history of epilepsy, and a lack of consent to participate in the study. The TML Rapid Test Strip (urine) was employed to confirm the presence of tramadol poisoning. Emergency Medical Services (EMSs) personnel and direct observation by the attending physician in the emergency room confirmed the occurrence of seizures through documentation. At the outset of the patient's admission, their blood glucose level was quantified via glucometer, and a blood sample was dispatched to the laboratory for examination of other biochemical factors. In the event of any deviation in blood glucose levels, the patient's blood glucose was reassessed using a glucometer at one-hour intervals. Demographic information, history of previous diseases, history of addiction, and previous poisoning with tramadol were asked from all patients and recorded in the checklist. The clinical symptoms and physical examination findings of the patients at the time of visit and also the vital signs of the patients at the time of visit and after 3, 6, and 24 h were examined. ECG was taken for all patients at the time of the visit. If an electroencephalography (EEG), brain CT scan and brain MRI were performed, and they were recorded in the prepared checklist. Biochemical tests including CBC, VBG, Na, K, Ca, P, CPK, BUN, Cr, BS AST, ALT, and ALP were also recorded in the prepared checklist. Data analysis was done by SPSS software Version 25. The relationship between quantitative variables was evaluated by the Spearman or Pearson correlation test (based on the distribution of variables in terms of normality). The relationship between qualitative variables was assessed by the Chi-square test. In the event of a non-normal distribution, equivalent nonparametric tests were employed. All tests were two-sided and the significance level was considered less than 0.05 (*p* < 0.05). All methods were conducted according to the relevant guidelines and regulations. Furthermore, the study was approved by the Mashhad University of Medical Sciences, Mashhad, Iran, and the Organizational Ethics Committee (IR.MUMS.MEDICAL.REC.1398.582).

## 3. Results

A total of 163 patients with an average age of 28.34 ± 9.36 years were studied, of which 138 patients (84.7%) were male. A history of addiction was present in 61% of the patients, the most common of which included cigarettes, tramadol, and opium, respectively. Tramadol poisoning had occurred in the past in 12% of the patients. The evaluation of the patient's vital signs at the time of admission showed that 25.3% of the patients had tachycardia (beats per minute 100 <) and 0.6% had bradycardia (beats per minute > 60). Tachypnea (breaths per minute < 20) was present in 5.1% of the patients, while bradypnea (breaths per minute 12 >) was not observed. The patient's body temperature was reported as normal. Systolic blood pressure was higher than 140 mmHg in 7.5% of the patients and lower than 80 mmHg in 1.3%.

Clinical symptoms and physical examination findings of the patients at the first time of visit are shown in Table 1. Of the total number of patients, 94 (57.3%) exhibited a reduced level of consciousness, while 69 (42.1%) experienced seizures. The ECG findings of the patients at the time of the visit are shown in Table 2. In 65 patients (39.6%), sinus tachycardia was observed. QT prolongation was observed in 28 patients. In addition, in 14% of the cases, the heart's axis exhibited a deviation to the left. In laboratory findings, 9 patients (13%) had CPK above 1000. Other investigated factors were reported within the normal range. CT scan and brain MRI were performed in 13 and 4 patients, respectively, and no pathological findings were observed in all cases. In addition, 11 patients underwent EEG, and the results were reported as normal in all cases.

The relationship between seizures in patients with their demographic variables and clinical symptoms is reported in Table 3. The results showed that the condition of the mucous membranes of the two groups was significantly different from each other, so in people with seizures, 44.8% had dry mucus and 7.5% had congested mucus, while in people without seizures, 30% had dry mucus and 20% had congested mucus (*p*=0.045). Also, limb pain was significantly higher in patients with seizures than in patients without seizures (*p*=0.021). In Table 4, vital signs at the time of the visit were compared between patients with and without seizures, and none of the vital signs were significantly different between the two groups with and without seizures (*p* > 0.05).  Table 5 presents the findings of the analysis conducted to examine the relationship between seizures in patients and their ECG findings. The results demonstrated that none of the patients' ECG findings exhibited a statistically significant correlation with the occurrence of seizures (*p* > 0.05). Laboratory findings were compared between patients with and without seizures. As can be seen in Table 6, the findings indicated that the mean ± standard deviation blood glucose levels of patients with seizures were significantly higher than those of patients without seizures (*p*=0.031), but there was no significant difference between the two groups in other cases. Furthermore, it was observed that among patients exhibiting blood glucose levels above 140 mg/dL, approximately 60% experienced seizures.

It should also be noted that 19.6% suffered acute kidney injury and were discharged after complete recovery. There were no deaths in our study.

## 4. Discussion

Drug poisoning is the most common poisoning in the world [22]. Narcotic drug poisoning, including tramadol, is particularly common among young people and contributes significantly to the overall number of poisoning cases [23]. This study examines the clinical and paraclinical signs and symptoms of patients with tramadol poisoning and their correlation with seizure occurrence. Farzaneh et al. evaluated 122 patients with tramadol poisoning, of whom 89.3% were male with an average age of 27 ± 7.2 years [24]. Also, in 2011, Eizadi Mood et al. investigated the clinical symptoms, duration of hospitalization, and outcome in tramadol poisoning patients; out of 184 patients, 141 were men with an average age of 24 ± 7 years [25]. The gender distribution of these studies is consistent with our findings.

Tramadol poisoning can result in a wide range of neurological complications, including seizures and a decreased level of consciousness. The onset of neurological effects occurs within the first 24 h following ingestion. Seizures are typically generalized tonic-clonic and occur within the first 6 hours [26]. The occurrence of seizures induced by tramadol appears to be dose dependent. Accordingly, higher doses of tramadol may be regarded as a risk factor [27]. Nevertheless, the occurrence of seizures has been documented even at doses considered therapeutic [2, 28]. In 2010, Borhani also investigated the incidence of seizures in patients with tramadol poisoning. The study demonstrated that tramadol has the potential to induce seizures in patients with and without a prior history of seizures. Moreover, the occurrence of seizures at therapeutic doses indicates that the tramadol dose is not a significant predictor of seizure occurrence [29]. Consequently, due to the idiosyncratic nature of tramadol in the occurrence of symptoms, an evaluation of its dosage was not conducted in the present study. Also, some studies have not identified notable discrepancies in serum tramadol concentrations between patients who experienced seizures and those who did not [2, 30, 31].

The Eizadi Mood study found that the most frequent reason for patients to be admitted to the hospital was a decreased level of consciousness (57%). Seizures (25%), tachycardia (25%), bradypnea (18%), and increased blood pressure (6%) were also common symptoms. Death occurred in 1.1% of the patients [25]. In our study, the most common symptoms reported by patients at the time of referral were convulsions and loss of consciousness. In our study, we did not observe any cases of death, unlike the study conducted by Izadi Mood et al. In addition, we found that symptoms such as sweating, tremors, clonus, impaired tendon reflexes, and limb pain were less frequent in our study patients. In patients who are addicted to this drug, not taking and prescribing naloxone can lead to deprivation syndrome, one of the symptoms of which is pain in the limbs. In addition, tonic-clonic seizures can cause joint damage and subsequent pain. Our study found that patients with seizures experienced more limb pain than those without. Tramadol may cause symptoms such as tachycardia and hypertension due to the inhibition of serotonin reabsorption [4]. Our study also observed a significant number of patients experiencing increased heart rate and blood pressure.

In 2013, Bushehri et al. conducted a study to investigate the clinical findings and acute complications resulting from tramadol poisoning in patients referred to a hospital in Urmia. The study included a total of 233 patients who had experienced tramadol poisoning. Of these patients, 78 (33.5%) experienced seizures and 154 (66.1%) experienced a decreased level of consciousness [32]. In this study, the rate of seizures was higher than that in Boushehri et al.'s study (42% vs. 33%). However, the decrease in the level of consciousness was observed in fewer patients in our study compared with Bushehri et al.'s study. The decrease in the level of consciousness in patients can be caused by an overdose of tramadol or due to the postictal phase of seizures. Both studies showed that the majority of patients recovered and there were no deaths observed, indicating consistency in their results.

In 2017, Golightly et al. conducted a retrospective study on 126 patients in the United States to investigate tramadol overdose symptoms. The reported symptoms included malaise, nausea, vomiting, tachycardia, restlessness, seizures, coma, hypertension, and respiratory depression. The study findings suggest that tramadol overdose primarily affects the nervous system rather than the cardiovascular system [33]. In our study, the ECG results showed that the most common rhythm was sinus rhythm, followed by sinus tachycardia, and no dangerous arrhythmias were observed. In addition, a large number of patients exhibited symptoms such as decreased levels of consciousness and seizures, indicating the effects of tramadol on the central nervous system.

In a study conducted by Moridani and Farhadi in 2017, it was reported that ECG and heart rate variability of patients could assist in predicting the occurrence of seizures. In addition, the greater and more straightforward accessibility of ECG evaluation renders it a potential alternative to EEG testing [19]. Similarly, a study conducted by Ufongene et al. (2020) revealed that an array of cardiac biomarkers, including abnormal QTc intervals, ST segment abnormalities, prolonged T waves, elevated P wave dispersion, and PR intervals, could be indicative of an increased risk for seizure occurrence [20]. In contrast to the findings of the aforementioned studies, our results demonstrated that there was no statistically significant correlation between ECG data and the occurrence of seizures.

In the study of Ghamsari et al. in 2016, which looked at the changes in ECG parameters in 1400 patients with tramadol poisoning, the results of the ECG rhythm of the patients in this study were similar to our study as normal sinus and then sinus tachycardia. However, unlike our results, the heart axis in the patients evaluated in this study shifted to the right [34].

Previous studies have shown that tramadol poisoning can lead to acute kidney failure [10, 35]. Similar to these studies, in our study, 19.6% of the patients had acute kidney injury. According to the CPK above 1000 units per liter of these patients, this complication occurred after rhabdomyolysis. In addition, it has been suggested that nephrotoxicity may occur not only with tramadol overdose but also with therapeutic doses [36].

Another adverse effect of a tramadol overdose is hypoglycemia [16, 37]. In diabetic rats, the activation of μ-opioid receptors by tramadol has been observed to increase glucose utilization and decrease hepatic gluconeogenesis [11]. In addition, some studies have documented the occurrence of hyperglycemia [14, 15]. It seems plausible to suggest that the monoaminergic pathways that contribute to the analgesic effect of tramadol may also be responsible for the hyperglycemia observed in some cases. It is, therefore, important to monitor blood glucose levels in patients with tramadol intoxication to manage any hypoglycemia or hyperglycemia and to avoid the complications that may arise from these conditions [13].

Our study found no significant relationship between the clinical manifestations of patients at the time of referral and the development of seizures. However, we did find a significant relationship between seizures and blood glucose levels, which were higher in patients with seizures than in those without. Currently, there are no reported studies on the correlation between elevated blood glucose levels and the incidence of seizures in individuals who have been poisoned with tramadol. Huang et al. conducted a study on hyperglycemia and seizures in diabetic patients. The results showed that hyperglycemia can increase the occurrence of seizures by creating a focal ischemic lesion in the brain. Therefore, blood glucose control is important in preventing seizures [38]. Another study on rats with diabetes demonstrated that elevated blood glucose levels decrease the seizure threshold and increase nervous excitability and seizures [39]. In a further analogous study by Schwechter et al., it was demonstrated that the administration of 40 mg/kg streptozocin to healthy mice resulted in the induction of hyperglycemia, which in turn led to a reduction in the seizure threshold. Consequently, as blood glucose levels increased, the incidence of seizures increased significantly [40].

According to the studies conducted so far, it has been determined that tramadol poisoning can lead to a decrease and increase in blood glucose in patients, although the mechanism of these changes has not been clearly stated and requires further investigation. However, the effectiveness of increasing blood glucose levels in the occurrence of seizures in these patients is probable. Finally, based on the results of this study and comparison with similar studies in this field, it can be concluded that tramadol poisoning is increasing among young people and there is a significant relationship between tramadol consumption and neurological symptoms, including the decreased level of consciousness and seizures. Due to the large-scale abuse of tramadol in recent years, further research must be conducted to investigate the clinical and laboratory symptoms associated with tramadol poisoning. This will aid in the prevention of complications and identify factors contributing to seizures in affected patients.

It should be noted that a limitation of this study is that the blood levels of tramadol were not assessed in patients. Consequently, it was not possible to investigate the potential relationship between tramadol levels and the occurrence of seizures. In addition, future research should assess HbA1c levels to ascertain a more precise causal relationship between tramadol use and alterations in blood glucose levels. A further limitation of our research is the lack of resources available for the comparison of results. Nevertheless, to the best of our knowledge, this is the inaugural study to report a significant correlation between the incidence of seizures and elevated blood glucose levels in patients with tramadol poisoning.

## 5. Conclusion

The majority of patients with tramadol poisoning had symptoms of reduced level of consciousness (57.1%) and seizures (42.1%). ECG findings had no significant relationship with the occurrence of seizures, and as a result, it cannot be a predictor of the occurrence of seizures. In the paraclinical examination of patients, a significant relationship between increased blood glucose levels and the occurrence of seizures was detected. Therefore, probably in the presence of hyperglycemia, patients will be at risk of seizures.

## Figures and Tables

**Table 1:** Clinical manifestations of patients at the time of visit.

Characteristic	Number	Percent
Loss of consciousness	94	57.3
Headache	50	31.3
Pupils		
Normal	132	81.5
Miosis	23	14.2
Mydriasis	7	4.3
Tremor	14	8.6
Clonus	4	2.5
Convulsions	69	42.1
Disorder of tendon reflexes	6	3.7
Mucous condition		
Normal	72	49
Dry	21	14.3
Congest	54	36.7
Limb pain	29	18.1
Sweating	64	39.5

**Table 2:** ECG findings of the patients included in the study.

Characteristic	Number	Percent
Rhythm		
Normal sinus	92	56.1
Sinus bradycardia	5	3
Sinus tachycardia	65	39.6
PVC + sinus tachycardia	2	1.2
Axis		
Normal	139	84.8
Deviation to the left	23	14
Deviation to the right	2	1.2
Long PR (> 0.2 s)	6	3.7
Short PR (< 0.12 s)	7	4.3
Wide QRS (> 0.12 s)	2	1.2
Prolonged QT (> 0.460 s)	28	17.3
Short QT (< 0.350 s)	12	7.4

**Table 3:** Correlation of seizures in patients with their demographic and clinical variables.

Characteristic	No seizures	With seizures	*p* value
Age (meaning ± standard deviation)	28.13 ± 8.42	28.62 ± 10.57	0.754
Gender, frequency (%)			
Male	80 (85.1)	58 (84.1)	> 0.999
Female	14 (14.9)	11 (15.9)	
History of addiction, frequency (%)	61 (64.9)	38 (56.7)	0.326
Previous history of tramadol poisoning, frequency (%)	9 (10.8)	8 (13.8)	0.609
Decreased level of consciousness, frequency (%)	51 (53.7)	43 (62.3)	0.337
Headache, frequency (%)	27 (29)	23 (34.3)	0.494
Pupils, frequency (%)			
Normal	73 (77.6)	59 (86.8)	0.213
Miosis	15 (16)	8 (11.8)	
Mydriasis	6 (6.4)	1 (1.4)	
Tremor, frequency (%)	9 (9.6)	5 (7.4)	0.779
Clonus, frequency (%)	1 (1.1)	3 (4.4)	0.310
Disorder of tendon reflexes, frequency (%)	2 (2.1)	4 (5.9)	0.239
Mucous state, frequency (%)			
Normal	40 (50)	32 (47.8)	0.045
Dry	24 (30)	30 (44.8)	
Congestion	16 (20)	5 (7.4)	
Limb pain, frequency (%)	11 (11.8)	18 (26.9)	0.021
Sweating, frequency (%)	31 (33.3)	33 (47.8)	0.074
Use of naloxone	31 (36)	18 (29)	0.383
Complete recovery, frequency (%)	45 (57)	24 (44.4)	0.163
Recovery with complications, frequency (%)	19 (24.1)	16 (30.2)	0.547

**Table 4:** Comparison of patient's vital signs at the time of visit between patients with and without seizures.

	With seizure, mean ± standard deviation or frequency (percentage)	No seizures, mean ± standard deviation or frequency (percentage)	*p* value
Body temperature (°C)	36.90 ± 0.25	36.89 ± 0.23	0.704
Pulses per minute	91.86 ± 17.82	91.43 ± 15.19	0.870
Breaths per minute	17.04 ± 1.83	16.93 ± 2.08	0.732
Systolic blood pressure (mmHg)	115.76 ± 14.56	117.18 ± 14.90	0.554
Diastolic blood pressure (mmHg)	73.61 ± 10.88	74.79 ± 11.48	0.515
Tachycardia	15 (23.1)	25 (26.9)	0.588
Bradycardia	1 (1.5)	0 (0)	0.411
Tachypnea	4 (6.3)	4 (4.3)	0.716
Bradypnea	4 (6.2)	8 (8.5)	0.580
Low blood pressure	1 (1.5)	1 (1.1)	> 0.999

**Table 5:** Comparison of ECG findings in patients with and without seizures.

Characteristic	With seizures	Without seizures	*p* value
Heart rate, mean ± SD	97.33 ± 22.43	94.16 ± 21.06	0.357⁣^∗^
PR interval, mean ± SD	0.14 ± 0.03	0.15 ± 0.03	0.130⁣^∗^
QRS duration, mean ± SD	0.07 ± 0.01	0.08 ± 0.07	0.328⁣^∗^
QTc interval, mean ± SD	0.41 ± 0.04	0.41 ± 0.07	0.439⁣^∗^
Long PR, frequency (%)	2 (3)	4 (4.3)	0.999 <⁣^∗∗∗^
Short PR, frequency (%)	3 (4.5)	4 (4.3)	0.999 <⁣^∗∗∗^
Wide QRS, frequency (%)	0 (0)	2 (2.1)	0.512⁣^∗∗∗^
Prolonged QT, frequency (%)	10 (14.9)	18 (18.9)	0.505⁣^∗∗^
Short QT, frequency (%)	2 (3)	10 (10.5)	0.071⁣^∗∗∗^
Rhythm, frequency (%)			
Normal sinus	36 (52.2)	56 (58.9)	0.452⁣^∗∗∗^
Sinus bradycardia	2 (2.9)	3 (3.2)	
Sinus tachycardia	31 (44.9)	34 (35.8)	
Sinus tachycardia + PVC	0 (0)	2 (2.1)	
Axis, frequency (%)			
Normal	59 (85.5)	80 (84.2)	0.477⁣^∗∗∗^
Deviation to the left	10 (14.5)	13 (13.7)	
Deviation to the right	0 (0)	2 (2.1)	

⁣^∗^An independent *t*-test was used to compare between two groups.

⁣^∗∗^Fisher's exact test was used to compare between two groups.

⁣^∗∗∗^The Chi-square test was used to compare between two groups.

**Table 6:** Comparison of laboratory findings of patients with and without seizures.

Laboratory marker	With seizures mean ± SD	No seizures mean ± SD	*p* value
PCO2 (mmHg)	50.52 ± 13.01	48.43 ± 11.08	0.298
pH	7.31 ± 0.09	7.33 ± 0.09	0.320
HCO3 (mEq/L)	24.79 ± 3.57	24.51 ± 4.76	0.690
Bexc (mEq/L)	−1.50 ± 3.62	−0.69 ± 4.05	0.221
Hemoglobin (g/dL)	14.71 ± 1.62	14.61 ± 1.92	0.730
Platelet (/L109)	241 ± 66	241 ± 62	0.982
WBC (/L109)	10.04 ± 3.77	9.95 ± 3.69	0.881
Na (mEq/L)	137.61 ± 3.50	138.07 ± 3.20	0.395
K (mEq/L)	3.98 ± 0.51	3.93 ± 0.43	0.545
Ca (mg/dL)	9.26 ± 0.52	9.20 ± 0.50	0.555
P (mg/dL)	4.12 ± 2.21	3.60 ± 1.32	0.417
Mg (mEq/L)	2.17 ± 0.31	3.48 ± 5.10	0.105
CPK (μ/L)	942 ± 1670	964 ± 3999	0.978
BUN (mg/dL)	28.29 ± 14.73	25.89 ± 8.79	0.199
Creatinine (mg/dL)	1.17 ± 0.46	1.08 ± 0.34	0.155
Blood sugar (mg/dL)	112.77 ± 68.51	92.36 ± 31.58	0.031⁣^∗^
AST (μ/L)	32.84 ± 41.60	28.45 ± 31.69	0.481
ALT (μ/L)	26.45 ± 24.33	25.66 ± 22.07	0.841
ALP (/L)	192 ± 67	178 ± 53	0.154

⁣^∗^*p* value is < 0.05 and significant.

## Data Availability

The data that support the findings of this study are available on request from the corresponding author. The data are not publicly available due to privacy or ethical restrictions.
